# Flexible material formulations for 3D printing of ordered porous beds with applications in bioprocess engineering

**DOI:** 10.1186/s40643-022-00511-9

**Published:** 2022-03-12

**Authors:** Simone Dimartino, Giuseppe Rafael Galindo-Rodriguez, Ursula Simon, Mariachiara Conti, M. Sulaiman Sarwar, Selva Manikandan Athi Narayanan, Qihao Jiang, Nick Christofi

**Affiliations:** 1 Institute for Bioengineering, School of Engineering, The University of Edinburgh, Edinburgh, EH9 3DW UK; 2 School of Applied Sciences, Edinburgh Napier University, Edinburgh, EH11 4BN UK

**Keywords:** Biomaterials, Additive manufacturing, Chromatography, Immobilized enzyme bioreactor, Bacterial biofilm bioreactor

## Abstract

**Background:**

3D printing is revolutioning many industrial sectors and has the potential to enhance also the biotechnology and bioprocessing fields. Here, we propose a new flexible material formulation to 3D print support matrices with complex, perfectly ordered morphology and with tuneable properties to suit a range of applications in bioprocess engineering.

**Findings:**

Supports were fabricated using functional monomers as the key ingredients, enabling matrices with bespoke chemistry, such as charged groups, chemical moieties for further functionalization, and hydrophobic/hydrophilic groups. Other ingredients, e.g. crosslinkers and porogens, can be employed to fabricate supports with diverse characteristics of their porous network, providing an opportunity to further regulate the mechanical and mass transfer properties of the supports. Through this approach, we fabricated and demonstrated the operation of Schoen gyroid columns with (I) positive and negative charges for ion exchange chromatography, (II) enzyme bioreactors with immobilized trypsin to catalyse hydrolysis, and (III) bacterial biofilm bioreactors for fuel desulphurization.

**Conclusions:**

This study demonstrates a simple, cost-effective, and flexible fabrication of customized 3D printed supports for different biotechnology and bioengineering applications.

**Supplementary Information:**

The online version contains supplementary material available at 10.1186/s40643-022-00511-9.

## Introduction

In the last decade we have witnessed the booming of additive manufacturing (AM, also 3D printing), including its related fabrication technologies, materials, and applications. The biotechnology and bioprocessing fields have been significantly influenced by AM, with reports spanning upstream and downstream processing, including sorting and selection of cell strains (Lin et al. 2016), bioreactors (Saha et al. 2018), harvesting (Shakeel Syed et al. 2017), filtration (Tan and Franzreb 2020), chromatography (Salmean and Dimartino 2019), and extraction (Wang et al. 2017). One of the most popular AM methods employed in bioengineering is digital light processing (DLP) where a three-dimensional model is built, layer upon layer, by selectively curing a photo-sensible liquid resin. Reasons for the success of DLP in biotechnology include its relatively low cost, fast speed (litre sized objects can be printed overnight), and high resolution (generally in the order of 50 μm or lower).

Historically, AM was primarily in the domain of the automotive, aerospace, and biomedical industries which favoured materials with mechanical properties over chemical characteristics and fabricated non-porous structures where strength and stiffness are key. On the other hand, bioprocess applications often require porous materials to maximize the total surface area available for cell adhesion, adsorption, and allow intraparticle mass transfer. Besides, materials in the biotechnology industry heavily exploit chemical characteristics, such as electrostatic charge and hydrophobic behaviour to appropriately modulate their interactions with species as diverse as cells, proteins, and small metabolites. This requirement contrasts the *status quo* where the composition of commercially available AM materials is IP protected, making it impossible to rationally design material–species interactions of interest. Proprietary compositions also complicate compliance with the Food and Drug Administration (FDA) or European Medicines Agency (EMA) requirements, hindering adoption of 3D printing in the biomanufacturing industry.

**Fig. 1:**
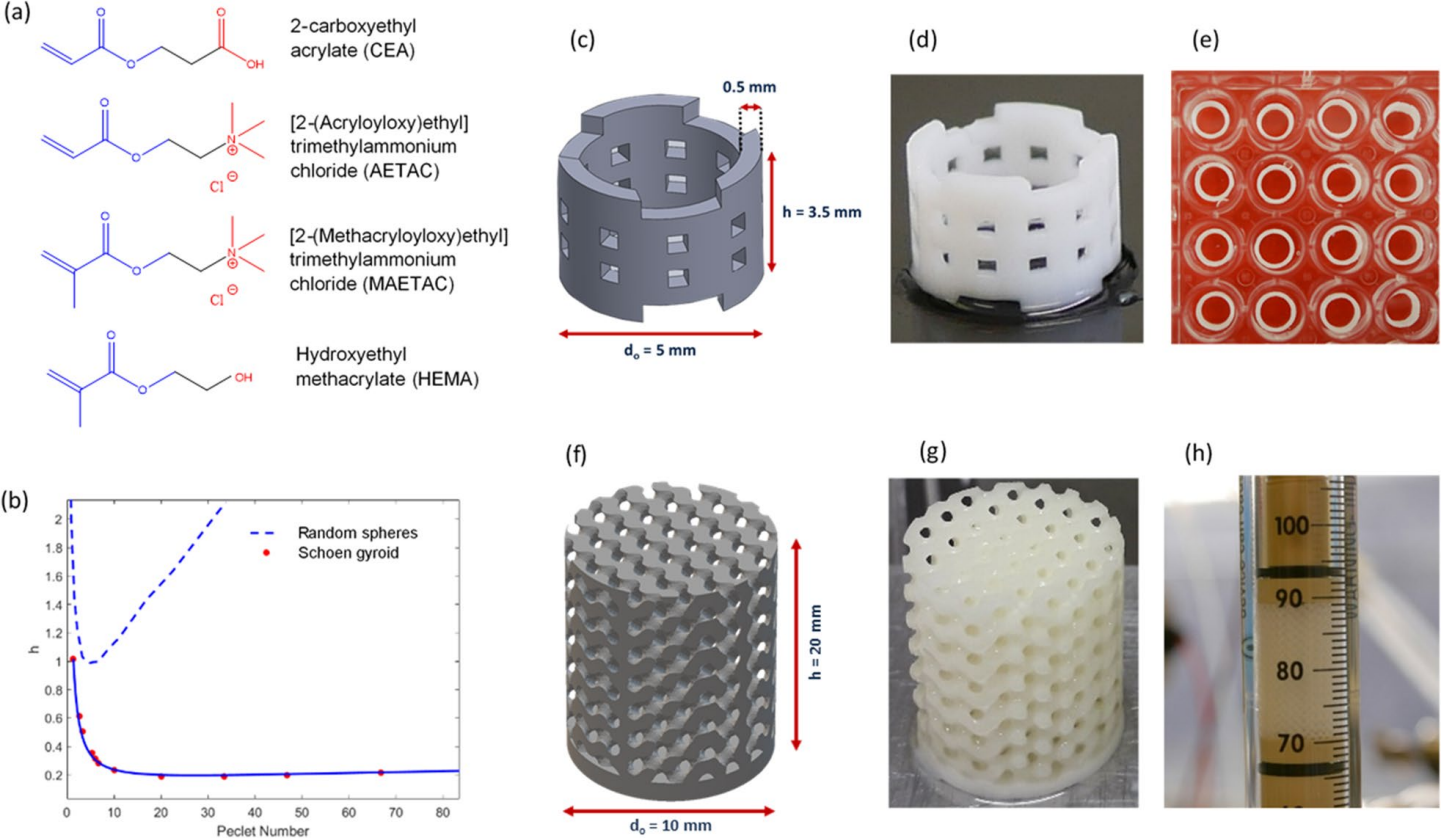
Schematic flow diagram for the design, manufacture, and experimental testing of porous monoliths for applications in bioprocess engineering. **a** Chemical structures of bifunctional monomers (CEA, AETAC, MAETAC, HEMA) employed, displaying (meth)acrylate groups for polymerization (blue) and active groups (red). **b** Simulated reduced plate height vs reduced velocity (Peclet) for the gyroidal scaffold (this work) and random packing of spherical particles (extracted from Schure et al. 2004). **c**, **f** CAD models of hollow cylinder and gyroid structures, respectively, together their 3D printed counterparts (**d**, **g**). Experimental characterization of **e** hollow cylinders using 96 multi-well plate set-up in static (batch) mode and **h** gyroid scaffold in column for dynamic testing

Here, we present a novel polymeric formulation for DLP 3D printing whose composition can be easily adjusted to alter the chemical and porous properties of printed parts. The formulation consists of a few simple ingredients, including monomers and crosslinkers to create the polymeric network (Fig. 1a), a UV photoinitiator to trigger the photopolymerization reaction, a photoabsorber to increase the resolution of the printed model, and porogenic components. The key feature of the proposed formulation lies in the use of bifunctional monomers bearing both a (meth)acrylate functionality for photopolymerization and a suitable chemical moiety for biomolecular interactions, e.g. charged groups, alkyl or aryl groups, or reactive groups for successive covalent immobilization of a desired ligand. By appropriate selection of the bifunctional monomers, materials with a range of surface derivatizations to suit a range of applications in bioprocess engineering can be obtained. Furthermore, the nature and relative concentration of the components making up the overall formulation will directly impact on the propagation kinetics of the free-radical polymerization reaction, in turn affecting the morphology of the resulting polymeric network and its porous microstructure (e.g. surface area, average pore size, pore size distribution) (Barner-Kowollik et al. 2014; Buback 2009).

In this work, we manipulated these characteristics to produce various formulations for DLP printing, and obtain porous monoliths with distinct chemical and macroporous properties for use in chromatography, immobilized enzyme bioreactors, and biofilm bioreactors.

## Materials and methods

### Composition of material formulations

All chemicals employed are listed in the Supplementary Information (SI). All formulations included 48% v/v cyclohexanol and 12% v/v dodecanol as porogens, and 1% w/v Omnirad 819 as photoinitiator. A mixture of poly(ethylene glycol) diacrylate (PEGDA, 12% v/v) and alkoxylated pentaerythritol tetraacrylate (SR494, 12% v/v) as crosslinkers, and 0.125% w/v Tinuvin 326 as photoabsorber was employed for the acrylate formulations. The relative concentration of the monomers, namely [2-(acryloyloxy)ethyl]trimethylammonium chloride (AETAC) and 2-carboxyethyl acrylate (CEA), was varied to adjust the ligand density (0, 4, 8, 12, 16% vol), with di(ethylene glycol) ethyl ether acrylate (DEGEEA) as non-functional monomer to obtain a total monomer concentration of 16% vol. The methacrylate formulation was composed of [2-(methacryloyloxy)ethyl]trimethylammonium chloride (MAETAC, 12% v/v) and hydroxyethyl methacrylate (HEMA, 12% v/v) functional monomers, ethylene glycol dimethacrylate (EDMA, 16% v/v) crosslinker, and Tinuvin 326 (0.1% w/v) photoabsorber.

### Model design, fabrication, and characterization

Computer-Aided Design (CAD) models of hollow cylinders and gyroidal columns were created on Fusion 360 (Autodesk, USA), exported as Standard Tessellation Language (STL) files, and sliced using Netfabb 2017 (Autodesk, USA). A Solflex 350 (W2P Engineering, Austria) DLP printer was employed to fabricate all parts. Post-printing, the parts were washed three times in isopropyl alcohol in an ultrasonic bath (Allendale Ultrasonics, UK) and then fully cured in water with a xenon Otoflash G171 unit (NK-Optik, Germany). The parts were stored in sterile 0.1 M phosphate buffer until use. A TM4000Plus scanning electron microscope (SEM, Hitachi, Japan) and a Zeiss Crossbeam 550 focused ion beam (FIB) SEM (Jena, Germany) were used for imaging, with samples prepared by freeze fracturing with liquid nitrogen and drying in ethanol, followed by a final wash in hexamethyldisilazane before sputter coating using an Emscope SC500 (Bio-Rad, UK). Mean pore sizes and distributions were evaluated from the SEM images (see section 2 in the Additional file 1 for additional details).

### Computational model and data analysis

Computational fluid dynamics was performed in COMSOL Multiplysics 5.4 (COMSOL, USA). A series of six gyroidal unit cells extending along the axial direction was built and the Navier Stokes equation for fluid motion was employed to determine the steady-state velocity field and estimate the pressure drop per unit length across the structure (no slip boundary conditions at the gyroidal walls, periodic boundary conditions at the cell periphery). The advection–diffusion model for mass transfer was then employed to model the dispersion of a pulse injection of an inert tracer (Schure et al. 2004). The average concentration profile over time at two different cross sections was employed to determine the plate height of the gyroidal column according to residence time distribution analysis (RTD) (Dolamore et al. 2018). Column permeability was calculated from the estimated pressure drop over the column.

### Chromatography

3D printed hollow cylinders were employed in batch experiments by inserting them into 96-well plates and reading the absorbance using a Modulus II microplate reader (Turner BioSystems, USA). Batch adsorption on the anion exchange chromatography (AEX) material (based on the AETAC monomer) involved an initial equilibration in phosphate buffer (20 mM, pH 7.4) for a minimum of 48 h, followed by addition of a bovine serum albumin solution (BSA, 0–32 mg/mL) in phosphate buffer. Similarly, cation exchange chromatography (CEX) materials (based on the CEA monomer) were equilibrated in binding buffer (20 mM phosphate, pH 7.4) before loading a lysozyme solution (LYS, 0–4 mg/mL). Flow experiments were carried out feeding protein mixtures of BSA, LYS, and myoglobin (MYO) onto gyroidal columns (50% external porosity, 500 µm wall thickness) slotted into 10 mm i.d. SNAP® glass housing (Essential Life Solutions, USA) and connected to an ÄKTA™ Purifier 10 system (GE Healthcare, Sweden) equipped with a UV detector to record absorbance at 280 nm. The flow rate was kept at 1 mL/min (75 cm/h linear velocity) as generally employed in lab-scale experiments.

### Immobilized enzyme bioreactor

Trypsin was immobilized on CEA supports via the 1-Ethyl-3-(3′-dimethylaminopropyl)carbodiimide (EDC) protocol. Briefly, the 3D printed materials were equilibrated in a 0.1 M sodium phosphate (pH 7.4) activation buffer, followed by a 35-min immersion on activation buffer containing 1:10 molar excess of EDC with respect to carboxylic groups. After extensive washing in activation buffer, coupling of the enzyme was obtained by soaking the 3D printed models in trypsin solutions (1–10 mg/mL) in 0.1 M phosphate buffer pH 7.4 for 2 h at room temperature. Non-bound trypsin was removed by washing with 0.1 mM Tris buffer (pH 8). The amount of trypsin immobilized on the 3D printed materials was calculated as the difference of the initial and final concentration of trypsin using the bicinchoninic acid (BCA) assay (Smith et al. 1985). A control experiment was run by adding trypsin solutions to non-activated cylinders. Similar to the chromatography runs, the activity of the immobilized trypsin was tested both in batch (hollow cylinders in multi-well plate format) and dynamic conditions (gyroids with 50% external porosity, 500 μm wall thickness, 25 mm diameter, 10 mm bed height, flow rate ranging from 0.5 to 8 mL/min). In both cases, after equilibration in 50 mM Tris buffer pH 8, a 1 mM N-α-benzoyl-l-arginine ethyl ester hydrochloride (BAEE) substrate solution in 50 mM Tris buffer pH 8 was fed to the 3D printed models, and formation of the N-α-Benzoyl-L-arginine (BA) hydrolysis product was monitored at 253 nm.

### Bacterial biofilm bioreactor

Biofilms of *Rhodococcus opacus* IEGM 248 cells were obtained by perfusing fresh cultures (exponential growth phase) for 3 days in recirculation mode (1 mL/min) through gyroidal supports (50% external porosity, 2 mm wall thickness, 10 mm diameter, 40 mm height) in a glass column, followed by column washes with quarter strength Ringer’s solution to remove non-adsorbed biomass (free cells). The obtained biofilms were then grown by continuous feed (2 mL/min) of a mineral salts medium (MSM, 2.0 g/L sucrose, 7 g/L Na_2_HPO_4_, 6 g/L KH_2_PO_4_, 2 g/L NH_4_Cl, 0.2 g/L MgCl_2_·6H_2_O, 0.03 g/L CaCl_2_·2H_2_O, 0.001 g/L FeCl_3_·6H_2_O) spiked with 0.2 mM benzothiophene (BT) as sole sulphur source. According to the biodesulphurization reaction, BT is converted into phenolic compounds (principally hydroxyphenylacetaldehyde) whose presence in the perfusate was confirmed using the Gibbs test (Gibbs 1927; Wang et al. 2013).

## Results and discussion

### Structure morphology at the macro and micro scales

A Schoen gyroid was selected for fabricating the structured monoliths (Fig. 1f, g). Gyroids are members of Triply Periodic Minimal Surfaces (TPMS), highly versatile geometries with maximized surface area for mass transfer (Femmer et al. 2015) and excellent load-bearing stiffness. TPMS are described by simple equations and their properties in terms of size, surface area, hydraulic diameter, bed porosity, tortuosity, wall thickness, etc., can be tweaked by altering the equation parameters (Schoen 2012). We first simulated fluid flow and solute dispersion to validate the suitability of the gyroid topology for packed beds. The gyroid structure showed fivefold higher efficiency (measured in terms of minimum reduced plate height, Fig. 1b) and fourfold higher permeability (6.4 × 10^–14^ m^2^) compared to traditional random packing of spherical particles (1.61 × 10^–14^ m^2^) (Schure et al. 2004). In particular, the interconnected gyroid lattice ensured appropriate radial intermixing, in turn reducing axial dispersion and band broadening, while showing lower flow resistance than random beds.

Use of porogens enabled formation of a highly interconnected porous network defined by polymeric globules (Additional file 1: Fig. S1). The final porous microstructure was principally determined by polymer chemistry, with acrylate-based formulations producing smaller pores (271 ± 120 nm for the AETAC and 289 ± 112 nm for the CEA materials, Additional file 1: Fig. S1d) than methacrylate-based formulations (905 ± 410 nm for the MAETAC material). This is consistent with the higher reactivity of acrylates over methacrylate groups (Barner-Kowollik et al. 2014), with acrylates generating a higher number of polymerization nuclei, in turn leading to smaller globules and smaller pores than methacrylates. Further tuning of the porous characteristics of the matrices could be achieved by precise adjustment of the composition of the liquid resin employed for the 3D prints, as its nature and concentration directly affects the thermodynamic properties and the polymerization kinetics of the mixture. This represents an open opportunity to ultimately enable modulation of the diffusional mass transfer properties within the 3D printed scaffold, the surface area available for adsorption and reaction, as well as structural and mechanical characteristics.

### Application as stationary phase for ion exchange chromatography

**Fig. 2:**
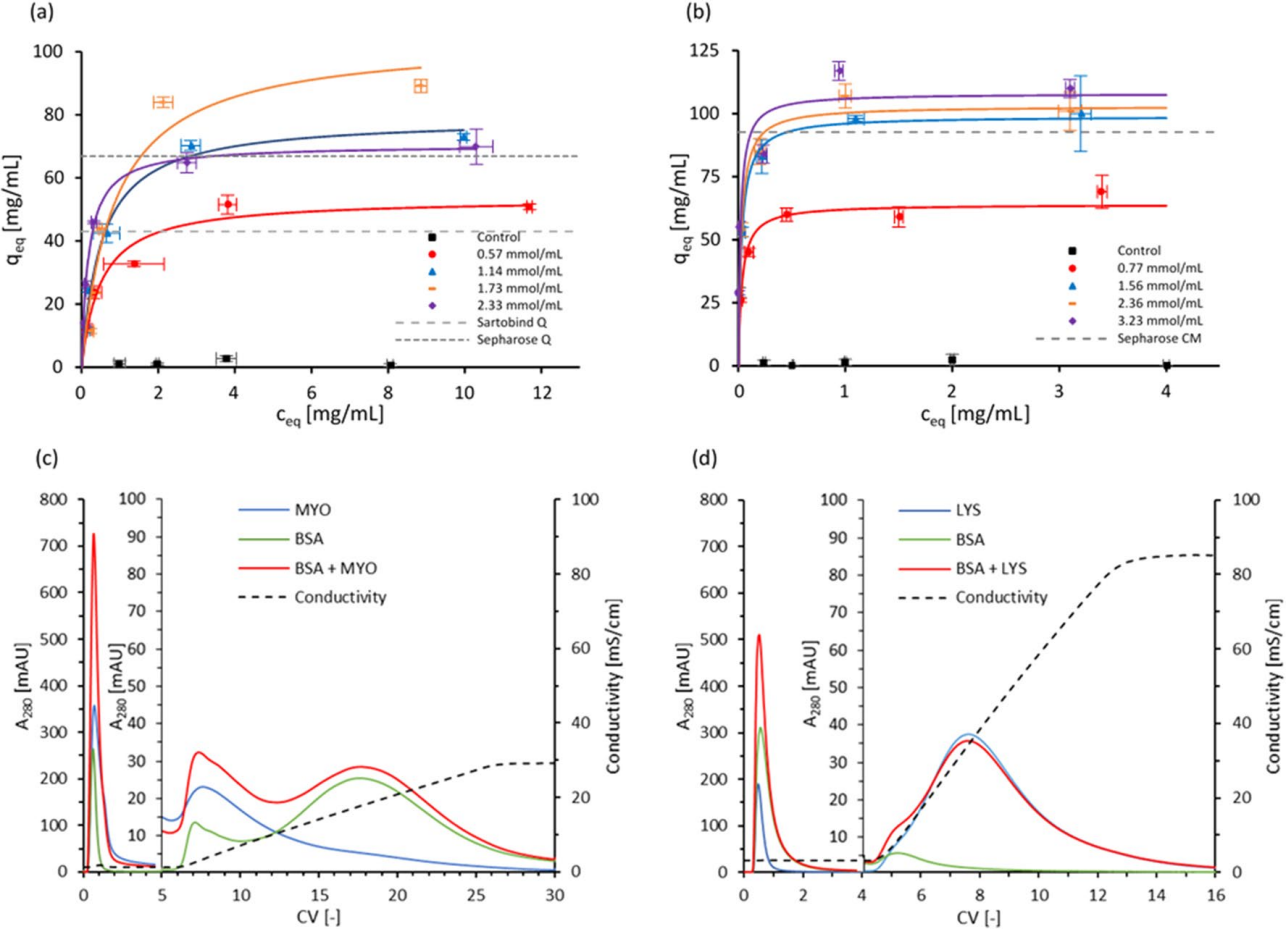
Adsorption isotherms of **a** BSA on anion exchangers (based on AETAC monomer, adapted from Simon et al. 2020), and **b** LYS on cation exchangers (based on CEA monomer). Ligand densities of 0 (control), 0.57, 1.14, 1.73, 2.33 mmol/mL and of 0 (control), 0.77, 1.56, 2.36, 3.23 mmol/mL were tested for the anion and cation exchanger, respectively. Isotherms were fitted with Langmuir model (continuous lines). Dashed lines correspond to maximum binding capacity of equivalent commercial materials (Boi et al. 2020; Staby et al., 2005). **c** Separation of BSA (16 mg/mL) and MYO (6 mg/mL) on AETAC-based anion exchangers (1.6 mL column volume (CV), 1.73 mmol/mL ligand density). 500 μL injection in binding buffer (20 mM Tris, pH 8.0) followed by linear gradient from 0 to 30% elution buffer (20 mM Tris, 1 M NaCl, pH 8.0) over 20 CV at 1.0 mL/min (75 cm/h). First peak is flow through of overloaded BSA and MYO; second and third peaks correspond to MYO elution at 7.4 CV (3.1 mS/cm) and BSA elution at 18.7 CV (19.0 mS/cm) in line with the electrostatic interactions established at the buffer’s pH of 8 (pI_BSA_ = 4.8; pI_MYO_ = 7.0). **d** Separation of BSA (16 mg/mL) and LYS (4 mg/mL) on CEA-based cation exchangers (2.5 mL CV, 3.23 mmol/mL ligand density). 300 μL injection in binding buffer (25 mM phosphate, pH 7.4) followed by linear gradient from 0 to 100% elution buffer (25 mM phosphate, 1 M NaCl, pH 7.4) over 20 CV at 1.0 mL/min (75 cm/h). First peak is flow through of non-binding BSA (pI_BSA_ = 4.8) and overloaded LYS; second peak corresponds to LYS elution at 7.6 CV (33.4 mS/cm) in line with the electrostatic interactions established at the buffer’s pH of 7.4 (pI_LYS_ = 11.4)

We first demonstrate use of the proposed formulation for chromatography applications. In particular, strong anion and weak cation exchange monoliths were fabricated using resins with bifunctional acrylates bearing positive quaternary amine (AETAC monomer) and negative carboxyl groups (CEA monomer), respectively (Fig. 1a). Ion exchangers bearing different ligand densities were obtained by altering the concentration of the functional monomers in the formulation, enabling adjustment, and optimization of the adsorption characteristics towards the target solute. The ion exchangers were initially 3D printed as hollow cylinders (Fig. 1c–e), and adsorption of pure BSA and LYS was measured in batch conditions on the AETAC and CEA materials, respectively. Maximum binding capacities of 104.2 ± 10.6 mg of BSA per mL of AETAC-based support and 108.1 ± 25.9 mg of LYS per mL of CEA-based material were recorded (Fig. 2a, b), about fivefold higher than for commercial monoliths (Hahn et al. 2002) and chromatographic membranes (Boi et al. 2020) and in line or above standard chromatographic resins (Staby et al. 2005). Testing in dynamic conditions was carried out using Schoen gyroid columns (Fig. 1f–h) by loading BSA and MYO onto the AETAC material (Fig. 2c, Simon et al. 2020) and BSA and LYS on the CEA material (Fig. 2d). The chromatograms reveal elution patterns in line with the electrostatic interactions established at the buffer’s pH, thus confirming the availability of the surface quaternary amine and carboxyl groups to establish appropriate electrostatic interactions with the protein models. Also, approximately 90% of the proteins adsorbed were recovered during elution for both materials, demonstrating that the strength of the electrostatic interactions can be appropriately adjusted to enable bind and elute operation of the ion exchangers. The broad elution peaks confirm mass transfer limitations from the bulk of the 3D printed media to the liquid phase during elution (Simon et al. 2020), an open challenge requiring improvements in the resolution achievable with current 3D printers. Appropriate process development also needs to be carried out to determine the optimal operating conditions to maximize the overall yield of the separation step.

### Application as immobilized enzyme bioreactor

**Fig. 3:**
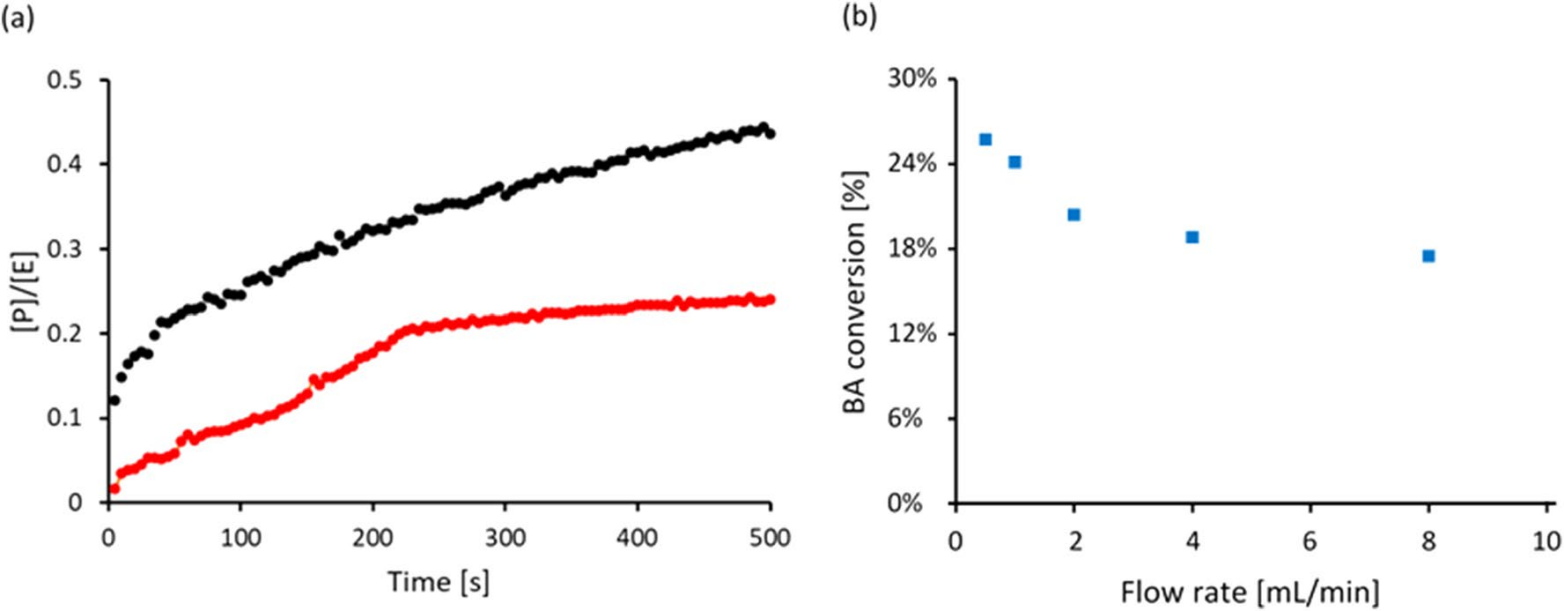
**a** Formation of the BA hydrolysis product in batch conditions (per unit concentration of Trypsin enzyme in the system) using 3D printed hollow cylinders with 7.31 mg/g immobilized trypsin (red) and using free trypsin in solution at a concentration of 2 mg/mL (black). **b** BA conversion obtained from continuous operation of immobilized enzyme bioreactor with Schoen gyroid bed geometry in steady-state mode at different flow rates. In both cases, the monoliths were equilibrated in 50 mM Tris buffer pH 8, followed by introduction of a 1 mM BAEE substrate solution (in 50 mM Tris buffer pH 8), with UV monitoring of the formation of the hydrolysis product (BA) at 253 nm

The potential to fabricate immobilized enzyme bioreactors for biotransformations was tested by covalently immobilizing trypsin onto the free carboxylic groups of the CEA material. In particular, 3D printed supports were exposed to trypsin solutions having different enzyme concentrations, leading to materials with progressively increasing immobilized trypsin, up to a maximum density of 7.5 ± 0.04 mg of trypsin per g of support, corresponding to 66% utilization of the theoretical carboxyl groups. Enzymatic activity was tested in batch experiments by introducing BAEE to 3D printed hollow cylinders, and monitoring the formation of the BA product from the enzyme catalysed reaction. Results indicated that the immobilized trypsin retained its hydrolytic activity (Fig. 3a), with an average loss of activity of 49.8% with respect to free trypsin in solution as frequently observed in the practice of immobilized enzymes (Guedidi et al. 2010). Dynamic experiments were carried out using 3D printed gyroids to demonstrate the operation of the bioreactor in steady-state, continuous mode. Five flow rates in the range of 0.5–8 mL/min were tested and product formation was verified for all of them. As the flow rate increased, the concentration of product in the effluent stream decreased due to a combination of mass transport phenomena and actual reaction kinetics (Fig. 3b).

### Application as bacterial biofilm bioreactor

**Fig. 4:**
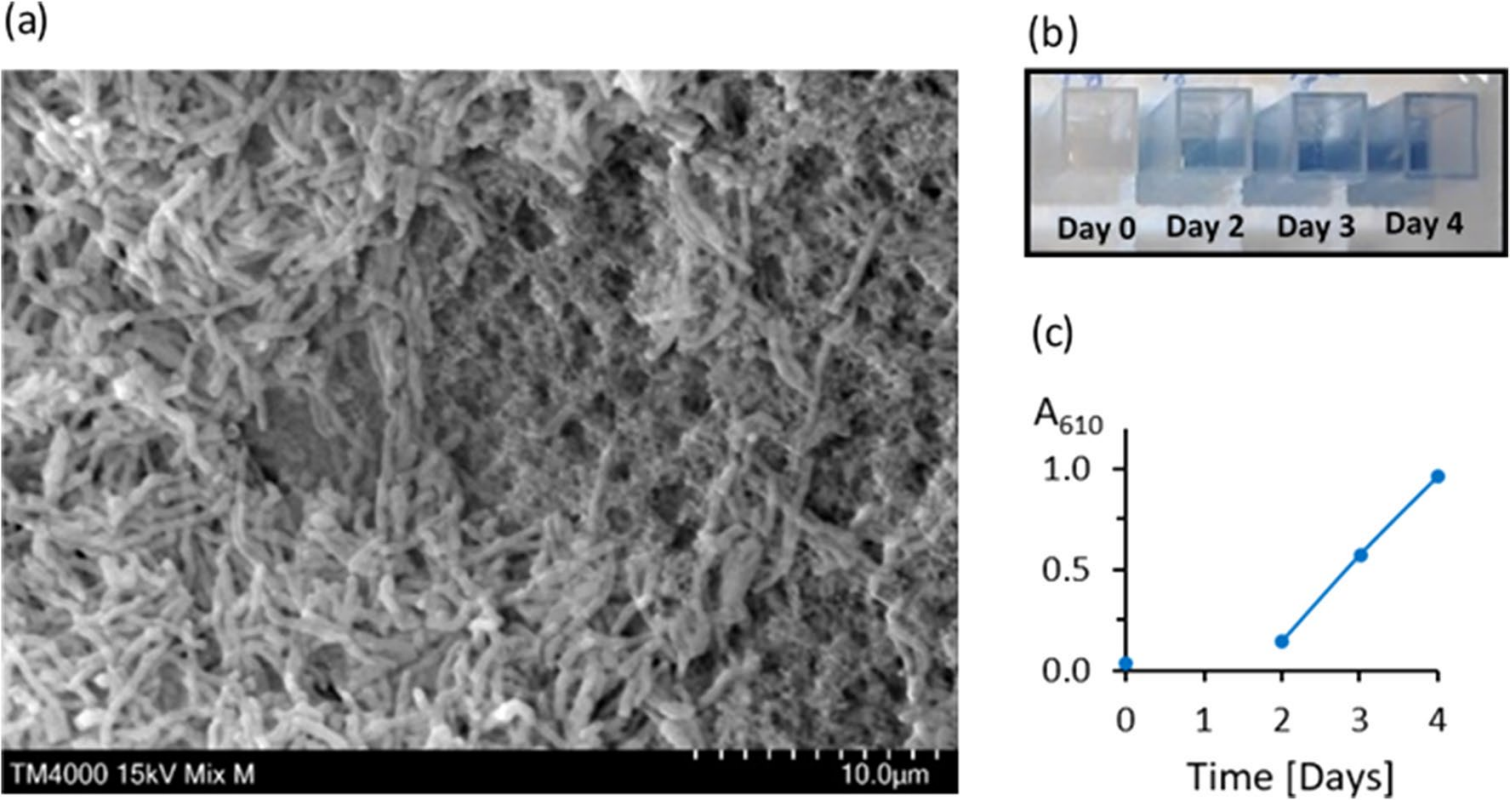
**a** SEM of MAETAC monomers revealing bacterial biofilm adhered onto 3D printed support after 3 days of incubation with *R. opacus* culture in exponential growth phase. BT is desulphurized by *R. opacus* into phenolic end products, as detected using Gibbs test on perfusate samples visualized by blue colour (**b**) and measured at 610 nm (**c**)

The bioreactor concept was extended to whole-cell biocatalysis. We selected the biodesulphurization reaction catalysed by immobilized *Rhodococcus opacus* as a case study for an emerging eco-friendly alternative to traditional desulphurization techniques employed in oil refineries (Mohebali and Ball 2016). *R. opacus* has negatively charged cell membrane rich in mycolic acid. Accordingly, the support material was engineered with positively charged and hydrophilic bifunctional monomers displaying quaternary amine (MAETAC) and hydroxyl groups (HEMA, Fig. 1a) to enhance cell immobilization. A methacrylate functionality was chosen to enable autoclaving of the supports for repeated use. Immobilization experiments with standard culture medium revealed that *R. opacus* bacteria formed stable and healthy biofilms on the 3D printed supports (Fig. 4a). In particular, a biofilm covering the external surface of the supports was obtained, with rod-like morphology typical of filamentous aggregates of mature *Rhodococcus* cells (approximately 4.0 and 0.5 microns in length and width, respectively). Since the pore size of the matrix is between 0.4 and 1.0 μm, the biofilm could penetrate into the porous architecture, aiding cell adhesion, and biofilm stability. The gyroid-based bioreactor was tested for the biodesulphurization of BT in perfusion mode. Results reveal BT conversion into phenolic end products (Fig. 4b, c), proving the biodesulphurization potential of the 3D printed bacterial biofilm bioreactor. In addition, production of phenolic compounds increases over time, indicating adaptation of the biofilm to the reacting conditions and confirming the biocompatibility of the support material with a thriving bacterial population.

## Conclusions

Taken together, the versatile material formulation presented here could be used to create complex three-dimensional matrices for chromatography and bioreactors. The chemical and microporous properties of the supports can be easily adjusted by changing the composition of the DLP resin formulation. For example, a combination of bifunctional monomers bearing alkyl chains and polar groups could be employed to alter the hydrophobicity/hydrophilicity as well as charged state of the matrices. In the future, precise control over the porous microstructure will also enable fine tuning of the mass transfer and mechanical characteristics of the supports. We expect this versatility, coupled with cost-effective and rapid DLP 3D printing (all models were 3D printed in a few hours), will truly enable fabrication of complex three-dimensional architectures to suit a range of diverse experimental requirements for a multitude of applications in bioprocess engineering.

To enable this objective, further material development, characterization and optimization is needed, e.g. stability and swelling properties of the 3D printed structures when exposed to different buffers, leakage of immobilized ligands, etc. Also, further efforts are needed on process development, optimization and validation to guide implementation of 3D printed supports in the research and the industry.

### Supplementary Information

**Additional file 1: Figure S1.** SEM images of (**a**) CEA, (**b**) AETAC and (**c**) MAETAC polymers together with their pore size distribution (**d**).

## Data Availability

All data generated and analysed during this study are included in this published article and its supplementary information file.

## References

[CR1] Barner-Kowollik C, Beuermann S, Buback M, Castignolles P, Charleux B, Coote ML, Hutchinson RA, Junkers T, Lacík I, Russell GT, Stach M, Van Herk AM (2014). Critically evaluated rate coefficients in radical polymerization-7. Secondary-radical propagation rate coefficients for methyl acrylate in the bulk. Polym Chem.

[CR2] Boi C, Malavasi A, Carbonell RG, Gilleskie G (2020). A direct comparison between membrane adsorber and packed column chromatography performance. J Chromatogr A.

[CR3] Buback M (2009). II. Fundamentals of free-radical polymerization propagation kinetics in radical polymerization studied via pulsed laser techniques. Macromol Symposia.

[CR4] Dolamore F, Fee C, Dimartino S (2018). Modelling ordered packed beds of spheres: the importance of bed orientation and the influence of tortuosity on dispersion. J Chromatogr A.

[CR5] Femmer T, Kuehne AJC, Torres-Rendon J, Walther A, Wessling M (2015). Print your membrane: rapid prototyping of complex 3D-PDMS membranes via a sacrificial resist. J Membr Sci.

[CR6] Gibbs HD (1927). Phenol tests: III. The indophenol test. J Biol Chem.

[CR7] Guedidi S, Yurekli Y, Deratani A, Déjardin P, Innocent C, Altinkaya SA, Roudesli S, Yemenicioglu A (2010). Effect of enzyme location on activity and stability of trypsin and urease immobilized on porous membranes by using layer-by-layer self-assembly of polyelectrolyte. J Membr Sci.

[CR8] Hahn R, Panzer M, Hansen E, Mollerup J, Jungbauer A (2002). Mass transfer properties of monoliths. Sep Sci Technol.

[CR10] Lin X, Yao J, Dong H, Cao X (2016). Effective cell and particle sorting and separation in screen-printed continuous-flow microfluidic devices with 3D sidewall electrodes. Ind Eng Chem Res.

[CR11] Mohebali G, Ball AS (2016). Biodesulfurization of diesel fuels—past, present and future perspectives. Int Biodeterior Biodegradation.

[CR12] Saha A, Johnston TG, Shafranek RT, Goodman CJ, Zalatan JG, Storti DW, Ganter MA, Nelson A (2018). Additive manufacturing of catalytically active living materials. ACS Appl Mater Interfaces.

[CR13] Salmean C, Dimartino S (2019). 3D-printed stationary phases with ordered morphology: state of the art and future development in liquid chromatography. Chromatographia.

[CR14] Schoen AH (2012). Reflections concerning triply-periodic minimal surfaces. Interface Focus.

[CR15] Schure MR, Maier RS, Kroll DM, Davis HT (2004). Simulation of ordered packed beds in chromatography. J Chromatogr A.

[CR16] Shakeel Syed M, Rafeie M, Henderson R, Vandamme D, Asadnia M, Ebrahimi Warkiani M (2017). A 3D-printed mini-hydrocyclone for high throughput particle separation: application to primary harvesting of microalgae. Lab Chip.

[CR17] Simon U, Scorza LCT, Teworte S, McCormick AJ, Dimartino S (2020). Demonstration of protein capture and separation using three-dimensional printed anion exchange monoliths fabricated in one-step. J Separat Sci.

[CR18] Smith PK, Krohn RI, Hermanson GT, Mallia AK, Gartner FH, Provenzano MD, Fujimoto EK, Goeke NM, Olson BJ, Klenk DC (1985). Measurement of protein using bicinchoninic acid. Anal Biochem.

[CR01] Staby A, Sanda MB, Hansenb RG, Jacobsen JH, Andersen LA, Gerstenberg M, Bruus UK, Jensena IH (2005) Comparison of chromatographic ion-exchange resins: IV. Strong and weak cation-exchange resins and heparin resins. J Chromatogr A 1069(1):65–77. 10.1016/j.chroma.2004.11.094 10.1016/j.chroma.2004.11.094 15844484

[CR19] Tan R, Franzreb M (2020). Continuous ultrafiltration/diafiltration using a 3D-printed two membrane single pass module. Biotechnol Bioeng.

[CR20] Wang W, Ma T, Lian K, Zhang Y, Tian H, Ji K, Li G (2013). Genetic analysis of benzothiophene biodesulfurization pathway of *Gordonia terrae* strain C-6. PLoS ONE.

[CR21] Wang H, Cocovi-Solberg DJ, Hu B, Miró M (2017). 3D-printed microflow injection analysis platform for online magnetic nanoparticle sorptive extraction of antimicrobials in biological specimens as a front end to liquid chromatographic assays. Anal Chem.

