# Supplementary material for: Flexible material formulations for 3D printing of ordered porous beds with applications in bioprocess engineering
Source: Bioresour Bioprocess. 2022 Mar 12;9(1):20. doi: 10.1186/s40643-022-00511-9 (PMC10992019; doi:10.1186/s40643-022-00511-9)
Supplement: Supplementary file 1 — Additional file 1: Figure S1. SEM images of (a) CEA, (b) AETAC and (c) MAETAC polymers together with their pore size distribution (d). [file 40643_2022_511_MOESM1_ESM.pdf]

SUPPLEMENTARY MATERIAL FOR

Flexible material formulations for 3D printing of ordered porous beds with applications in bioprocess engineering

Simone Dimartino[1,*], Giuseppe Rafael Galindo-Rodriguez[1], Ursula Simon[1], Mariachiara Conti[1], M. Sulaiman Sarwar[1], Selva Manikandan Athi Narayanan[1], Qihao Jiang[1], Nick Christofi[2]

[1] Institute for Bioengineering, School of Engineering, The University of Edinburgh, Edinburgh, EH9 3DW, UK

[2] Recircle Ltd, NINE BioQuarter, 9 Little France Road, Edinburgh, EH16 4UX, UK

1. Chemicals

[2-(Acryloyloxy)ethyl]trimethylammonium chloride (AETAC), 2-carboxyethyl acrylate (CEA), 2-Hydroxyethyl methacrylate (HEMA), [2-(Methacryloyloxy)ethyl]trimethylammonium chloride (MAETAC), di(ethylene glycol) ethyl ether acrylate (DEGEEA), hexamethyldisilazane (HMDS), Gibbs reagent (2,6 dichloroquinone-4-chloroimide) and TRIZMA HCl were purchased from Sigma-Aldrich. Cyclohexanol, 1- dodecanol, isopropanol (IPA), bovine serum albumin (BSA, protease-free powder), lysozyme, trypsin, sodium phosphate (mono and dibasic), N-α-benzoyl-L-arginine ethyl ester hydrochloride (BAEE) and Pierce™ bicinchoninic acid (BCA) assay kit were purchased from Thermo Fisher Scientific. 1-Ethyl-3-(3'-dimethylaminopropyl)carbodiimide HCl (EDC) was purchased from Novabiochem. The salts used in the preparation of mineral salts medium (MSM) were sourced from VWR Chemicals (GPR RECTAPUR® grade). Benzothiophene (BT) was purchased from Alfa Aesar, UK. The isotonic diluent Ringer's solution tablets were purchased from Oxoid, UK. Ethylene glycol dimethacrylate (EDMA), poly(ethylene glycol) diacrylate (PEGDA) and alkoxylated pentaerythrioltetraacrylate (SR494) were kindly donated by Arkema-Sartomer (Colombes,

France). Omnirad 819 was donated from IGM resins (Waalwijk, The Netherlands). Tinuvin 326 was donated from BASF (Ludwigshafen, Germany). All buffers were prepared using deionized water (EuRO10 Reverse Osmosis System, EvoquaWater Technologies, Pittsburgh, PA, USA).

2. Porous microstructure

The porous mictrostructure was determined through SEM images (Figure S1.a, b and c) following the protocol described by (Kalsoom et al., 2018) in which a set of 500 pores were measured in each SEM using ImageJ software, and finally computing their relative frequency (Figure S1.d). Note the polymeric network is determined by the polymeric globules, with both sharing similar characteristic dimension.

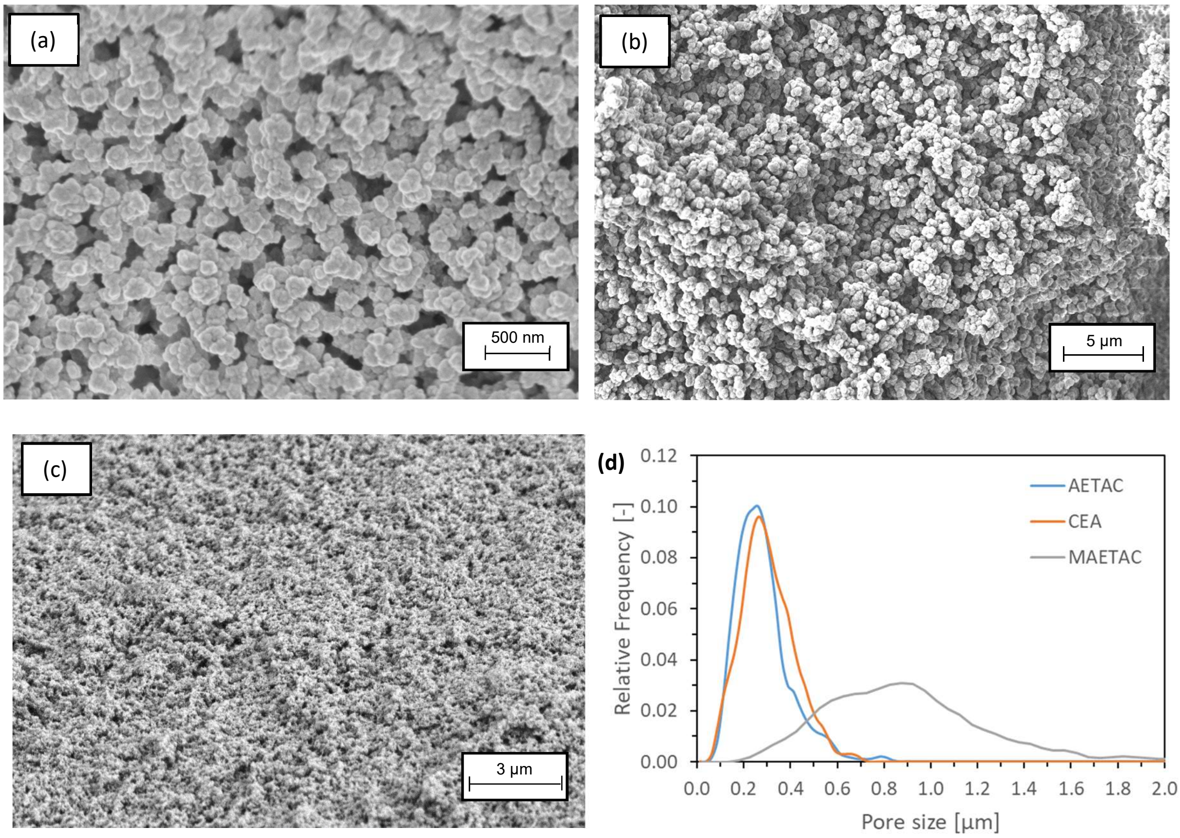

Figure S1. SEM images of (a) CEA, (b) AETAC and (c) MAETAC polymers together with their pore size distribution (d).

Kalsoom, U., Hasan, C. K., Tedone, L., Desire, C., Li, F., Breadmore, M. C., Nesterenko, P. N., & Paull, B. (2018). Low-Cost Passive Sampling Device with Integrated Porous Membrane Produced Using Multimaterial 3D Printing. *Analytical Chemistry*, *90*(20), 12081–12089.
